# Protocol for a feasibility study of group-based focused psychosocial support to improve the psychosocial well-being and functioning of adults affected by humanitarian crises in Nepal: Group Problem Management Plus (PM+)

**DOI:** 10.1186/s40814-018-0315-3

**Published:** 2018-07-18

**Authors:** Manaswi Sangraula, Edith van’t Hof, Nagendra P. Luitel, Elizabeth L. Turner, Kedar Marahatta, Jolene H. Nakao, Mark van Ommeren, Mark J. D. Jordans, Brandon A. Kohrt

**Affiliations:** 1 Transcultural Psychosocial Organization Nepal, Baluwatar, Kathmandu, Nepal; 2 Department of Mental Health and Substance Abuse, World Health Organization, Geneva, Switzerland; 3 Department of Biostatistics and Bioinformatics and Duke Global Health Institute, Duke University, Durham, USA; 4 World Health Organization, Country Office for Nepal, Kathmandu, Nepal; 5 Emergency Response and Recovery Branch, Division of Global Health Protection, Center for Global Health, Centers for Disease Control and Prevention (CDC), Atlanta, GA USA; 6 Office of U.S. Foreign Disaster Assistance (OFDA), United States Agency for International Development (USAID), Washington, DC USA; 7 Centre for Global Mental Health, Institute of Psychiatry, Psychology, and Neurosciences, King’s College London, London, UK; 8 Department of Psychiatry and Behavioral Sciences, George Washington University, Washington, DC USA

**Keywords:** Low- and middle-income countries, Mental health, Non-specialists, Group interventions, Humanitarian emergencies

## Abstract

**Background:**

The prevalence of common mental disorders increases in humanitarian emergencies while access to services to address them decreases. Problem Management Plus (PM+) is a brief five-session trans-diagnostic psychological WHO intervention employing empirically supported strategies that can be delivered by non-specialist lay-providers under specialist supervision to adults impaired by distress. Two recent randomized controlled trials in Pakistan and Kenya demonstrated the efficacy of *individually* delivered PM+. To make PM+ more scalable and acceptable in different contexts, it is important to develop a *group version* as well, with 6–8 participants in session. A study is needed to demonstrate the feasibility and acceptability of both the intervention in a new cultural context and the procedures to evaluate Group PM+ in a cluster randomized controlled trial.

**Methods:**

This protocol describes a feasibility trial to Group PM+ in Sindhuli, Nepal. This study will evaluate procedures for a cluster randomized controlled trial (c-RCT) with Village Development Committees (VDCs), which are the second smallest unit of government administration, as the unit of randomization. Adults with high levels of psychological distress and functional impairment will receive either Group PM+ (*n* = 60) or enhanced usual care (EUC; *n* = 60). Psychological distress, functional impairment, depression symptoms, posttraumatic stress disorder (PTSD) symptoms, and perceived problems will be measured during screening, pre-treatment baseline, and 7–10 days after the intervention. Qualitative data will be collected from beneficiaries, their families, local stakeholders, and staff to support quantitative data and to identify themes reporting that those involved and/or effected by Group PM+ perceived it as being acceptable, feasible, and useful. The primary objective of this trial is to evaluate the acceptability and feasibility of the intervention; to identify issues around implementation of local adaptation methods, training, supervision, and outcomes measures; and to assure that procedures are adequate for a subsequent effectiveness c-RCT.

**Discussion:**

Outcomes from this trial will contribute to optimizing feasibility and acceptability through cultural adaptation and contextualization of the intervention as well as refining the design for a c-RCT, which will evaluate the effectiveness of Group PM+ in Nepal.

**Trial registration:**

ClinicalTrials.gov identifier: NCT03359486

## Background

Humanitarian crises, such as the earthquake in Nepal in April 2015, cause significant psychological and social suffering. Nepal’s fragmented and under-resourced mental health and social services are not able to cope with such a high level of need [1]. The country has 0.22 psychiatrists and 0.06 psychologists per 100,000 people, mainly working in large cities [1]. Nepal has basic health care units with primary care staff and midwives, and in most districts, there are other community care providers, often working for NGOs. The availability of this system makes a model of care provision through non-specialists a particularly important implementation strategy.

In low-resource settings, mental health interventions may need to be short of duration and carried out by non-specialists in the communities to make them sustainable and feasible to implement on a broader scale. A simplified psychological intervention, *Problem Management Plus* (*PM+*), has been developed by the World Health Organization (WHO) to address this. It has four core features that make the intervention suitable for low-resource settings exposed to adversities: a brief intervention (five sessions) (1) delivered individually or in groups; (2) delivered by non-specialists (high school graduates with no mental health experience), using the principle of task shifting; (3) designed as a trans-diagnostic intervention, addressing a range of client-identified emotional (e.g., depression, anxiety, stress) and practical problems; and (4) designed for people in communities in low- and middle-income countries (LMIC) affected by any kind of adversity (e.g., violence, disasters) [2].

Recent randomized controlled trials (RCTs) in Peshawar (Pakistan) and Nairobi (Kenya) have indicated individually delivered PM+ to be effective in diminishing depression and anxiety symptoms, managing self-selected practical or psychological problems, and improving daily functioning [3, 4]. The first evaluation of a Group PM+ is underway in Pakistan [4]. This paper describes the study protocol of a feasibility trial with Group PM+ in Nepal before evaluating effectiveness in a fully powered cluster RCT (c-RCT) [5]. Feasibility studies are valuable to address issues related to process, resources, management, or scientific approaches [6, 7] in so the issues can be addressed before conducting definitive randomized trials.

### Objectives

For Group PM+ in Nepal, we will implement trial procedures to gather information about feasibility, acceptability, safety, and delivery of the intervention in a Nepali community setting, and to assess training, supervision, and outcomes measures. The Group PM+ manual has been adapted for post-earthquake rural Nepal through qualitative formative research (has not been published). The feasibility trial will further identify whether the clinical and content adaptations are appropriate for the setting. Possible problems of acceptability, compliance, delivery of the intervention, randomization, blinding, recruitment, and retention will be assessed before the effectiveness c-RCT is conducted [7]. The feasibility study will include two trial arms: enhanced usual care (EUC) and Group PM+. We will assess the acceptability and feasibility of the Group PM+ intervention compared to EUC and will collect data for the design of a full-scale effectiveness c-RCT of Group PM+ compared to EUC. We will use a mixed-methods design with qualitative and quantitative approaches to determine feasibility. The objectives include the following:

1. To evaluate the feasibility and acceptability of the Group PM+ intervention in a rural Nepal community [primary objective];
2. To evaluate the feasibility and acceptability of intervention delivery by Group PM+ trained non-specialists;
3. To determine recruitment and retention rates for Group PM+ sessions;
4. To assess feasibility and acceptability of outcome measures;
5. To assess feasibility of cluster randomization procedure to limit biases and risk of contamination;
6. To assess ethics and safety of trial procedures using the adverse event protocol.

## Methods/Design

### Setting

Nepal is a low-income country in South Asia with a population of approximately 27 million with the majority (83%) of the population living in rural areas [8]. The country suffered a decade-long civil war from 1996 to 2006 with a range of psychiatric sequelae among adults and children [9–11]. In 2015, there were two major earthquakes in 2015, killing approximately 10,000 people and injuring 20,000. A mental health epidemiological study in Sindhupalchowk, Gorkha, and Kathmandu conducted 3 months post-earthquake found that one in three adults were experiencing depression and anxiety, one in five adults engaged in harmful alcohol use, and one in ten adults had current suicidality [12]. The compromised infrastructure and limited availability of specialized mental health services is an impediment to addressing this burden of mental health problems.

The study will take place in Sindhuli district, a region southeast of Kathmandu, which was heavily impacted by the earthquakes. In Sindhuli, 250 people were injured and 15 were killed. Over 22,000 households were fully damaged and 10,000 partially damaged. In response to the earthquake’s effects on Sindhuli, Transcultural Psychosocial Organization (TPO) Nepal in collaboration with International Medical Corps (IMC) conducted mental health and psychosocial support (MHPSS) activities in over half of the district’s Village Development Committee (VDCs) from 2015 to 2017. TPO Nepal is a Nepali non-governmental mental health research and training organization, with specific expertise in humanitarian settings [13]. For the Group PM+ feasibility study, two Village Development Committees (VDCs) that had not previously received services were selected for randomization to either EUC or the intervention. Approximately 5000 people live in each VDC.

The selected VDCs have a diverse population with over 15 ethnicities, including Brahman/Chhetri, Magar, Tamang, and Dunwar. The national language Nepali is spoken by the majority of inhabitants. A formative qualitative study in these VDCs demonstrated that residents of these VDCs have minimal access to and awareness of mental health issues and its treatment. Each VDC has one government health post, which represents the first and most accessible portal of care, though often not the well-resourced. Primary healthcare workers in these facilities include health assistants, community medical assistants, auxiliary nurse midwives, and female community health volunteers (FCHVs).

### Design

Randomization will occur at the VDC level, and one VDC will receive the intervention while the other will receive EUC. Though not identical, the two VDCs are similar in population size, ethnic demographics, and access to health facilities. The two VDCs will be randomized in a public drawing by the District Public Health Officer (additional details provided below in the *Randomization* procedure). The two VDCs are separated by an adjoining VDC in attempt to limit intervention contamination among the beneficiary populations. Because there are two units of analysis for this trial, adjustment for clustering will be considered for analyzing the effectiveness of the definitive trial.

### Intervention: EUC versus Group PM+ intervention

Until recently, treatment-as-usual in rural Nepal for individuals with common mental disorders (CMD) in Nepal usually consists of no psychological/psychiatric treatment in local health facilities. Whereas experiencing a CMD rarely leads to treatment initiation, persons with severe mental illnesses are typically brought by family members to tertiary psychiatric services in the Kathmandu valley, and this is often after a long delay between onset of symptoms [1]. Beginning in 2012, the WHO mental health Gap Action Programme (mhGAP) Intervention Guide was adapted for use in Nepal and piloted in Chitwan district through the Programme for Improving Mental Health Care (PRIME) [14]. After the 2015 earthquakes, the mhGAP Humanitarian Intervention Guide [15] was adapted and contextualized for Nepal, and Nepali psychiatrists were taught to train primary care workers using mhGAP. This approach was used in Sindhuli. Therefore, the EUC arm in Nepal will receive a referral to primary care-based depression treatment.

Participants in the Group PM+ arm will receive five 3-h sessions of Group PM+. Each session focuses on teaching participants’ techniques to manage their stressors and problems. These sessions include (1) managing stress, (2) behavioral activation, (3) managing problems, (4) strengthening social support, and (5) review of techniques [16]. See Table 1 for more details on each session.

**Table 1:** Mechanisms of action of Group PM+ intervention

PM+ mechanisms of action	Description of mechanism	Implementation of mechanism
Stress management	Participants learn deep breathing. They are encouraged to incorporate this mechanism into daily life (i.e., when doing housework, walking, etc.). Grounding techniques are incorporated to bring participants back to the present.	Session 1
Behavioral activation	Participants review the inactivity cycle. They choose a small activity that they enjoy doing (i.e., making and drinking tea, meeting a friend, etc.) and create a detailed plan about when and how to conduct this activity as a first step in breaking the inactivity cycle.	Session 2
Managing problems	Participants learn which of their problems are solvable and which are unsolvable. One problem is chosen among the solvable problems, and participants brainstorm tangible solutions, then creating manageable steps to accomplish their goals.	Session 3
Strengthening social support	Participants learn to recognize who among their family and friends are existing and potential sources of support and how best to strengthen connections with them. Social network mapping activities are incorporated in this mechanism.	Session 4

Note: The first four sessions of PM+ each addresses a specific mechanism of action. The fifth and last session is a review of the mechanisms of actions learned in the previous sessions

There will be a total of 60 participants in each arm. In the intervention arm, there will be approximately 7–10 groups with six to eight participants per group, separated by gender and with gender-matched facilitators. Facilitators will be supported by volunteer helpers in organizing the logistics of the group sessions, reminding participants about the sessions, and meeting non-attenders (participants who do not show up for Group PM+ sessions). Participants will be provided with calendars and reminder calls by the facilitators’ helpers, if necessary, to decrease dropout rates.

To conduct awareness-raising activities and facilitate recruitment, five non-specialists will be recruited in the EUC arm and another five in the Group PM+ arm. The requirement for the non-specialists will be at least 10 years of education, over 25 years of age, and living in either the EUC or Group PM+ VDC. The non-specialists will be trained by TPO Nepal for 20 days on basic psychological skills to become community psychosocial workers (CPSWs). Twenty days is the standard length for CPSW training through TPO Nepal, based on the expectation that briefer training would not equip facilitators to provide quality care to intervention participants. CPSWs from the intervention arm will then be given a 10-day Group PM+ training using the adapted manual and other clinical materials. Intervention training includes education on adversity and its impact upon mental health, basic counseling skills, delivering Group PM+, skills in group facilitation, and facilitator self-care. Group “Helpers” will receive a basic 2-day training on assisting facilitators during Group PM+ sessions and participating alongside CPSWs in practice PM+ groups. The main role of helpers will be logistics and child care. Competency and fidelity will be assessed with modified version of the Enhancing Assessment of Common Therapeutic Factors (ENACT) tool tailored for Group PM+ [17].

### Feasibility criteria

The primary objective is to evaluate feasibility and acceptability of both the intervention and the trial procedures for the subsequent c-RCT through the collection of both quantitative and qualitative data [4]. Because feasibility and acceptability are complex domains, both quantitative and qualitative indicators will clarify what procedures to carry on to the full trial and where modifications should be made to study design or content [18]. The following quantitative indicators will determine progression to the main trial:

- *Fidelity to Group PM+ elements at the level of 75% or greater* according to the mean fidelity checklist for Group PM+ elements across all sessions;
- *Lack of significant socio-demographic group differences*; tabulation of descriptive summaries for baseline characteristics comparing Group PM+ participants and EUC participants without significant group differences in education, economic status, age, gender, and medical comorbidities;
- *Retention of at least 67% of participants* through completion of five Group PM+ sessions;
- *Fewer than 15% missing items* on outcome measures across all assessments;
- *Presence of adverse events among fewer than 10%* of participants and any serious adverse events;

The following qualitative indicators will determine progression to the main trial:

- *Identification of qualitative themes* reporting that both CPSWs and beneficiaries perceive Group PM+ as being acceptable, feasible, and useful; the qualitative data will be coded for themes that participation reduces psychological distress, that participation does not damage familial or community relations, that participation is perceived as safe, and that participation is not perceived as stressful resulting in worsening mental health (see Table 2);

**Table 2:** Qualitative domains and objectives

Domains	Participants interviewed	Sample research questions
1. Acceptability of Group PM+	Participants, family, CPSWs, community, psychosocial team	Is PM+ stigmatizing? Is it acceptable for CPSW to deliver PM+? What were parts of the program that could have been changed to make the program more acceptable for the community?
2. Implementation logistics; PM+ sites, local leadership	CPSWs, community, RAs and research staff, psychosocial team	How would we enhance project implementation (in terms of venue, coordination with local leadership, etc.)?
3. Feasibility of PM+ and burden (time, frequency, distance for providers and participants)	Participants, family, CPSWs, community, psychosocial team	How would make this program more sustainable? How would make this program more effective? Should the program be longer?
4. Fidelity and supervision (areas of deviation and cause, competency, amount and form of supervision)	CPSWs, psychosocial team	How did the CPSWs deviate from the material in the PM+ manual? Why did they deviate from the material? Was there a need for more or less supervision? What were the challenges to supervision?
5. Utility (perceived benefit) of PM+	Participants, family, CPSWs, community, psychosocial team	How do CPSWs perceive participant experience? What problems is PM+ helpful for? What problems is PM+ not helpful for? Who is PM+ useful for?
6. Contagion	Participants (control group), family, CPSWs, RAs and research staff, psychosocial team	Did anyone involved in PM+ teach friends, family, and community members PM+ techniques? Did the mechanisms of action for PM+ reach the control VDC? If so, how did those in the control group learn?
7. Blinding/randomization; sources and timing of unbinding	CPSWs, mhGAP, community, RAs and research staff, psychosocial team	When did RAs and CPSWs know that different groups received different treatment? How did they know about the different groups?
8. Recruitment and retention (participants and providers)	Participants, family, CPSWs, community, RAs and research staff, psychosocial team	What were challenges to recruitment? What were challenges to retention of participants in the program? What are possible solutions to recruitment and retention?
9. Adverse events, ethics, safety	Participants, family, CPSWs, mhGAP, RAs and research staff, psychosocial team	Were staff equipped to handle any adverse events? What was the type and fBMW80481requency of adverse events referred?
10. Referral and control condition	Participants, family, CPSWs, mhGAP, community, psychosocial team	Were mhGAP services available? Was medication available in local health posts? Was the TPO counselor used by the community? Was transportation to local referrals available to those who needed it?
11. Assessment feasibility, acceptability, interpretation	Participants, RAs and research staff, CPSW	Were the assessments feasible to conduct? Did the participants understand the assessments? What were the challenges to conducting assessments?

Feasibility and acceptability will be evaluated by these indicators to determine progression to the full trial. In domains where criteria are met, we will retain the procedure for the full trial. In domains where criteria are not met, we will modify procedures for the full trial. The presence of any adverse events and serious adverse events will be addressed by the trial team to identify alternative strategies for the full trial and Data Safety Monitoring Committee, which is described in detail below. The number of feasibility and acceptability criteria that are not met will determine the extent of intervention and trial design modification.

### Measures/outcomes

Because the primary objective of this trial is to evaluate feasibility and acceptability, we will assess whether the established feasibility criteria were met. To support the five quantitative criteria listed above, qualitative data will be collected from beneficiaries, their families, local stakeholders, and staff to identify qualitative themes reporting that those involved and/or effected by Group PM+ perceived it as being acceptable, feasible, and useful. Qualitative interviews will be conducted throughout the trial (see Table 3).

**Table 3:** Qualitative interview schedule

Stakeholder	Definition	Type of interview	When
Beneficiaries/clients	Intervention (*n* = 10): participants who attended sessions regularly, those who dropped out sessions, those who improved in clinical outcomes at their follow-up assessment, those who did not improve in their clinical outcomes at follow-up, and at least one male from each of these sub-groups Control (*n* = 5): participants who improved in clinical outcomes at their follow-up assessment, those who did not improve in their clinical outcomes at follow-up, and at least one male from each of these sub-groups	Key Informant Interviews (KIIs)	After sessions of PM+ (*Rolling*)
Family	Intervention (*n* = 5): family members of participants who dropped out of sessions, family members of participants who did not (and did not) improve in their clinical outcomes at follow-up, and at least one male participant’s family from each sub-group Control (*n* = 3): family members of participants who did not (and did not) improve in their clinical outcomes at follow-up and at least one male participant’s family from each sub-group	KIIs	5 weeks after family meeting (*Rolling*)
CPSWs and helpers	Intervention (*n* = 4): CPSWs that facilitated sessions and helpers that assisted CPSWs in these sessions Control (*n* = 4): CPSWs	KIIs, Focus Group Discussions (FGDs)	After each session of PM+ training, during sessions, post-intervention
MhGap providers	Intervention and control (*n* = 2): Health workers in the local health posts that received training in mhGap	KIIs	After completion of intervention
Community Leaders	Intervention and control (*n* = 8): Community leaders (including members of mothers’ groups, local government officials, traditional healers, etc.) who received CIDT training as a part of recruitment efforts	KIIs	After completion of intervention
RAs and research staff	Intervention and control (*n* = 8): RAs and Research Supervisor	KIIs, FGDs	After completion of intervention
Psychosocial staff	Intervention and control (*n* = 3): Clinical Supervisors and counselor	KIIs, FGDs	After completion of intervention

Though the clinical outcomes in this feasibility and acceptability trial are secondary, the ability to measure them and have fewer than 15% missing items is a feasibility outcome. Clinical outcomes among participants will be measured through baseline (*t*_0_) and follow-up (*t*_1_) assessment. The baseline (*t*_0_) assessment will be conducted after the family meeting. The follow-up assessment (*t*_1_) will be scheduled 1–1.5 weeks after the fifth Group PM+ session (i.e., 8–8.5 weeks after the pre-intervention assessment). All instruments will be administered by trained research staff blind to the allocation status of the participants. The main analysis metric will be differences in primary and secondary outcomes between *t*_0_ and *t*_1_.

The primary clinical outcome measure will be the Patient Health Questionnaire (PHQ-9), a well-known 10-item instrument measuring symptoms of depression [19] (see Table 3). The measure has been clinically validated in Nepal [20]. There are eight secondary clinical outcome measures. To diminish the burden of time and questionnaires administered to the participants, many short-form versions of the assessments will be used. The WHO Disability Assessment Scale (WHODAS) has been used previously in Nepal [21–23], with excellent internal consistency between items (*α* = 0.90) and validity with multiple mental health measures for depression (*r* = 0.70, *p* < 0.001), anxiety (*r* = 0.64, *p* < 0.001), and PTSD (*r* = 0.37, *p* < 0.001). The GHQ-12 measures general psychological distress and has been clinically validated in Nepal [24]. The Psychosocial Mental Health Problems (PMHP) scale is a locally developed five-item assessment of common psychosocial problems [10]. The heart-mind screener is also locally developed and will be used to determine the acceptability of local idioms of distress and impairment due to these problems [20]. The PCL-5 (eight items) was shown in a recent study to have comparable diagnostic utility to the 20-item PCL-5 [25].

The Multidimensional Scale of Perceived Social Support (MSPSS) has been locally adapted in Nepal during a study among widows [26] and has been modified to for this trial. In the assessment, participants will assess their own connectedness with close family, friends, and other forms of support. The Reduced Tension Checklist (RTC) has been locally developed based on a coping checklist [27] to assess skill acquisition of PM+ skills. The Psychological Outcomes Profiles instrument [28] will be administered pre- and post-intervention as well as from sessions two to five for the PM+ intervention arm. The PSCYHLOPS will not be administered during session one of PM+ because of the proximity in time between pre-intervention and start of the sessions.

### Randomization

**Fig. 1:**
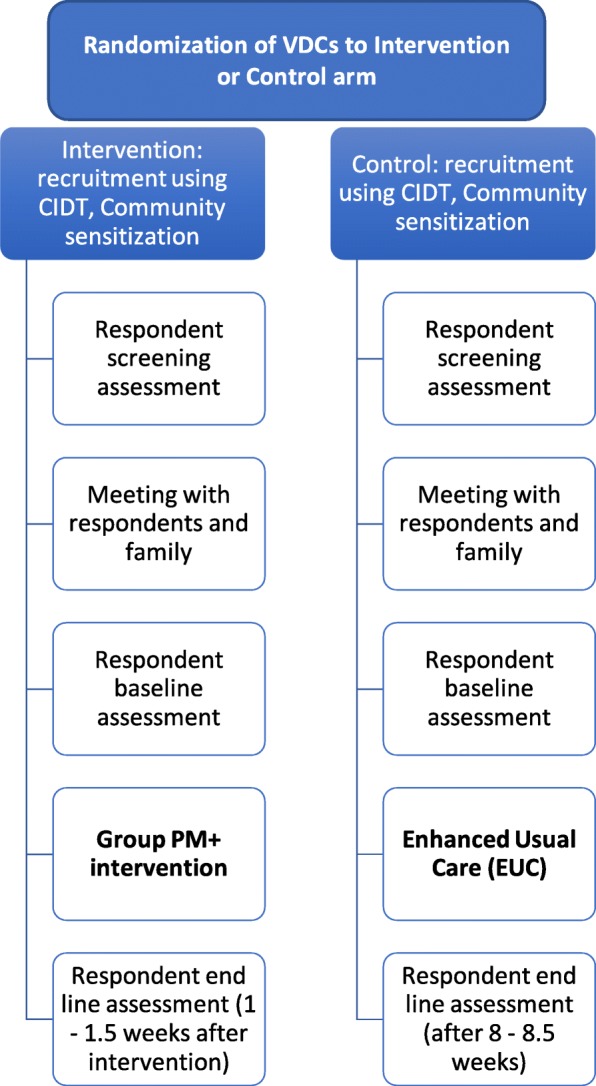
Flowchart for Group PM+ cluster randomized controlled trial. Flow diagram from recruitment to end line assessment for participants/respondents in control and intervention VDCs. Gray box represents intervention. Abbreviations: CIDT, Community Informant Detection Tool (see the “Recruitment” section). VDC, Village Development Committee

Two VDCs will be selected within Sindhuli district for the control and intervention arms (see Fig. 1). A meeting will be organized with the District Public Health Officer (DPHO) where VDCs will be randomly drawn for either of the trial arms. We chose to involve the DPHO in the randomization process to increase community engagement and governmental support for the research trial. The DPHO will conduct a drawing open to government staff and supervisors in the research team. CPSWs and RAs will not be present for this drawing. The DPHO will draw one of the two names out of a hat. There are several sources of potential contamination. CPSWs from both VDCs will be trained together for the initial 20-day community psychosocial training. Because of the proximity between the two VDCs, communities may be in contact with one another. CPSWs and RAs will be given a strict code of conduct to keep patient treatment confidential during the trial to reduce unblinding. Regardless, sources of potential contagions will be monitored closely and addressed in the full-scale trial after completion of the feasibility trial.

### Participants

Residents of the two VDCs 18 years of age and older are eligible for enrollment. There is no maximum age for the enrollment. However, assessors will use their discretion to discontinue screening for those that are unable to properly comprehend the questions due to age or are unable to physically reach session locations within the VDC.

#### Inclusion criteria

Adults potentially with a common mental disorder are eligible to participate when they are over 18 years old and speak and understand Nepali. The General Health Questionnaire (GHQ; see below) and the WHO Disability Assessment Schedule 2.0 will be used for the screening criteria. *Screening positive* is defined as positive on all the following: score > 2 on a screening questionnaire for common mental disorders [29, 30] and score > 16 on a screening questionnaire for functional impairments [31]. Because of the lack of other services and potential benefit from participation in Group PM+, individuals with suicidality are not excluded. However, persons with current suicidal plans will be referred to the TPO counselor in addition to the invitation to participate in Group PM+.

#### Exclusion criteria

Alcohol dependency will be assessed by the alcohol use disorders identification test (AUDIT). Persons with a score of 16 and higher will be excluded from participation. WHO’s guidelines for use in primary care report that people that score below 16 can benefit from simple advice [32] and also stated that people who score 16 and higher would benefit most from simple advice plus brief counseling and continued monitoring. For this reason, potential participants who score 16 and above on the AUDIT will be excluded from the study and referred to a mhGAP-trained health professional in the area. In case of any suspected severe psychiatric disorders (e.g., psychosis) or problems (e.g., active suicidality), the individual will be referred to the health facility where health workers have been trained in mental health treatment (following mhGAP) and/or the TPO counselor in the area. For urgent treatment (e.g., active suicidality), participants will be immediately referred to the local TPO counselor and/or the nearest psychiatric services, which are available in a hospital 7 h drive from the study site. A TPO Nepal counselor and clinical supervisor for the trial will also be available to facilitate the referral process and provide follow-up psychosocial care, if and when needed. Symptoms of psychosis and severe cognitive impairment are based on clinical judgment of the assessor. The assessor (research assistant) will be given training on a community case-finding tool for detection of psychosis [33], so they can better understand clinical symptoms for exclusion (see more details below on the community case detection tool in the “Recruitment” section). If the respondent is not able to comprehend or answer the consent and/or demographic questions coherently, the questionnaire will be terminated at that point.

In addition to collecting trial outcomes, we will conduct a qualitative component. We will conduct key informant interviews (KII) and focus group discussion (FGD) and collect process notes. For the qualitative component, we will select a subsample of intervention and control arm participants for KIIs and focus group discussions. In addition, we will conduct KIIs with CPSWs, family members of the participants, research staff, community officials, and primary health care staff.

### Recruitment

In the study VDCs, CPSWs will conduct awareness-raising activities to educate the public about availability of treatment for CMDs. In addition, female community health volunteers (FCHVs) and members of local community organizations (such as mothers’ groups, youth groups, etc.) will be trained on the Community Informant Detection Tool (CIDT) to identify people in the community with potential common mental disorders. The CIDT is a vignette-based tool for pro-active case detection by lay people, which has been developed and tested in Nepal [34]. The CIDT has a positive predictive value of 0.68 for adults [33]. The adapted version of CIDT for this study will include both inclusion vignettes (e.g., general distress, developed for the trial) and exclusion vignettes (e.g., psychosis, which have already been developed and validated). When community members and FCHVs identify a person in the community with symptoms of common mental disorders as described in the vignettes, they will ask them if they would like support for their stress-related problems. If people indicate they would like to receive support, then they will be told that a research assistant (RA) will visit them to conduct further screening. Individuals who meet CIDT criteria for exclusion conditions will be referred to local mhGAP-trained health workers. RAs will conduct additional recruitment by screening patients attending primary health care centers.

After screening by the RAs, CPSWs will hold a family meeting with the potential participant and a family member if they choose to have a family member participate. The family meeting will consist of (a) information about the results of the screening, (b) brief psychoeducation about the psychological consequences of adversity, and (c) information on seeking services from local health facilities with health care providers trained in basic mental health and psychosocial support. Those in the Group PM+ arm will also receive information about the intervention. Based on the family meeting, individuals can choose whether or not they want to enroll in their respective treatment arms and continue in the study.

### Blinding and concealment

CPSWs, RAs, trial participants, and local mhGAP-trained health workers will be blinded to the conditions of the two arms. Facilitators in the intervention arm and CPSWs in EUC will be instructed not to disclose the treatment that any participants are receiving except with their clinical supervisors. Assessors will be asked at baseline to indicate what treatment they think each participant will receive. Assessors will be asked the same question at end line for each participant. This will provide some data on the amount of unblinding that might occur in the RCT. Study statisticians will be blinded to treatment arm during analysis.

### Sample size

Approximately 60 participants will be enrolled in each treatment arm through pro-active case-finding methods. Approximately 60 participants were enrolled for each arm in a previous Group PM+ feasibility trial [5]. Because power calculations will not be carried out for this trial, 60 participants, or 7–10 groups, per arm will provide enough relevant information to inform feasibility and acceptability for the definitive effectiveness RCT following the trial. In addition, approximately 15 trial participants will be recruited for the qualitative interviews, as well as 15–18 additional key informants from the community. We also anticipate conducting qualitative interviews with research and psychosocial staff.

### Financial incentives

Participants will receive compensation in the form of household goods (e.g., soap, toothpaste) equivalent to 100–200 Nepali Rupees per assessment, to compensate for time invested in the research. Assessments will take a maximum of 1 h and 30 min, and participants will be informed of this time frame as part of the consent process. Participants will not be compensated monetarily for the time they spend in the sessions. For those in the treatment arm, snacks and tea will be offered to the participants at every session. Travel costs to sessions and to assessments will be compensated for as well. Actual cost basis is not currently feasible because of the unavailability of local transport receipts but a fixed amount for compensation will be calculated based on the area that they come from.

### Data management and monitoring

All principal investigators (PI) on the study will have access to primary data. The site PI will conduct quality assurance checks on data collected by the research assistants who will use a password-protected tablet to collect data. The data on the tablet will be synchronized and uploaded in the Open Data Kit (ODK) daily, saved on a private server, and transferred to a data-analytic computer program (e.g., SPSS) without the identifying key. Results will be published regardless of being negative or positive results and submitted to peer-reviewed scientific journals. A Data Safety Monitoring Committee (DSMC) will be established specifically for oversight of the trial and review of serious adverse events and adverse events. The DSMC will include psychiatrists, non-governmental organization experts in psychosocial programs, and researchers, and will determine any appropriate action in respect to ongoing trial conduct (e.g., referral to specialized care). The DSMC has the right to unblind at the individual level at any time.

### Planned analyses

#### Qualitative analyses

Focus group discussions (FGDs), key informant interviews, and process evaluation notes will be coded in NVIVO [35] and analyzed using content analysis [36] for themes of cultural acceptability, experience of CPSWs delivering Group PM+, adequacy of training duration, structure of training, content of training, and follow-up engagement, following approaches used in similar global mental health studies [37]. Coding will be done by multiple independent raters, and inter-rater reliability will be calculated using Kappa scores. Data analysis will be conducted throughout each step to facilitate iterative revision then finalization of the manual. Following the Consolidated Criteria for Reporting Qualitative Studies (COREQ), we will document the process according to the 32-item checklist [38]. Broadly, for domain 1 “research team and reflexivity,” the qualitative research team will include the PIs and TPO staff; the degrees will range from MD, PhD, to MA and Bachelors; the occupations will include academic medical faculty, NGO staff, and members of WHO; there will be both male and female qualitative staff; staff experience in qualitative research will range from 1 month to greater than 10 years; the relationship with participants will not precede the study; participants will know that research staff are employed by or associated with TPO Nepal; and interviewer characteristics (age, education, region of origin, etc.) will be reported. For study design, content analysis will be used; selection will be reported as described above; setting features including location and presence of non-participants will be reported; an interview guide will be used; there will be repeat interviews at different times in the training and supervision timelines; audio will be recorded; duration will be documented; data saturation or lack thereof will be reported; and transcripts will not be returned to participants for analysis. There will be approximately four coders; the coding tree will be published; themes will be identified in advance with the option to generate additional themes; participants will not provide feedback on the coding; quotations will be presented; data and findings will be consistent; and major and minor themes will be clearly presented.

#### Statistical analyses

We will employ statistical analyses comparable to those used in another pilot c-RCT being conducted in Nepal [39]. The quantitative outcomes of interest (Table 4) will be summarized descriptively using appropriate summary statistics (mean and standard deviation for continuous outcomes and numbers and proportions for categorical outcomes) and graphically over time for both study arms. Trends for each score will be plotted to examine between- and within-person differences and to determine the plausible pattern (e.g., linearity) of those trends. As noted by Eldridge et al., there are concerns that sample size estimates based on this trial’s data could be too small; therefore, we will also draw upon other studies in Nepal to inform the subsequent effectiveness study sample size [40]. We plan to power the full trial based on conservative estimates of the parameters of interest rather than exclusively those obtained from this c-RCT by using the upper bound of the 95% CI for the intra-class correlation coefficient (ICC) and by comparing our estimates to those from other studies of similar outcomes to be sure we will increase our estimates if we find them to be considerably smaller than those from other studies. By using such a “triangulation” approach and by obtaining context-specific data, we are confident that we will be able to better design the full-scale c-RCT than in the absence of the feasibility c-RCT data. The data will also be used to inform the choice of effect estimate (e.g., difference in slopes or in means at a specific follow-up time point) in the future c-RCT that will build on the current study. Preliminary indicative estimates of differences in primary and secondary outcomes by arm will be obtained. In practice, we will power the future c-RCT predominantly based on magnitudes of effect that are of public health relevance rather than using magnitudes of effects obtained from the study, which will not necessarily be indicative of what could be attained in an appropriately powered larger c-RCT.

**Table 4:** Quantitative outcome measures

Construct	Instrument	Description	Assessment time periods		
			Enrollment (−*t*_1_)	Baseline (*t*_0_)	Follow-up (*t*_1_)
Primary outcome (participants)					
Depression symptoms	Patient Health Questionnaire (PHQ-9)	Participants rate depression symptoms over past 2 weeks		X	X
Secondary outcomes (participants)					
Daily functioning	WHODAS	Participants rate their ability to engage in daily activities	X		X
General psychological distress	General Health Questionnaire(GHQ-12)	Participants measure their general psychological distress	X		X
General psychological distress	Somatic symptoms of Nepali Psychosocial and Mental Health Problems (PMHP)	Participants rate their somatic symptoms related to psychosocial health		X	X
General psychological distress	Heart-mind	Participants note if they have had any “*man ko samasya*” or heart-mind problems recently	X	X	X
General psychological distress	Tension Checklist	Participants note if they have had any tension recently		X	X
Alcohol use disorder	Alcohol Use Disorders Identification Test (AUDIT)	Participants rate alcohol use and associated behavior, as well as daily ethanol consumption	X		
Post-traumatic stress symptoms	PTSD Checklist for DSM5 (PCL-5)	Participants rate their post-traumatic stress symptoms on a scale		X	X
Personalized outcome	Psychological Outcome Profiles (PSYCLOPS)	Participants list their emotional and practical problems and rate how much each problem affects them		X	X
Additional measures of mechanisms and potential mediators					
Ways of coping	Reducing Tension Checklist (RTC)	Participants assess their own behavioral and psychosocial skills related to coping		X	X
Traumatic events	Traumatic Events Inventory (TEI)	Participants rate if they have been exposed to certain traumatic events throughout their lifetime		X	X
Perceived social support	Multidimensional Scale of Perceived Social Support (MSPSS)	Participants assess their own connectedness with close family, friends, and other forms of support		X	X
Suicidality	Suicidality	Participants rate if they have recently had suicidal thoughts, ideation, and plans	X		

#### Mixed methods framework

This feasibility study will follow the Good Reporting of A Mixed Methods Study (GRAMMS) guidelines: First, mixed methods are being used to evaluate feasibility and acceptability qualitatively while quantitative information will be used for the design of the full trial. Second, qualitative and quantitative will be assessed generally during the same intervals of the study after delivery of Group PM+. Both methods will be clearly documented in publications with regard to sampling, data collection, and analysis. Integration will occur in regard to qualitative descriptions of and quantitative scores on key variables. Because this is a feasibility study, inference testing on the quantitative data are limited; therefore, we cannot compare qualitative and quantitative data with regard to effectiveness of the Group PM+. Sixth, insights resulting specifically from integration of qualitative and quantitative will be highlighted.

### Ethics and research governance

#### Consent

The informed consent process will consist of two steps: informed consent for screening and informed consent for taking part in the Group PM+ trial. A research assistant will conduct informed consent for screening. When a possible participant screens positive, the CPSW will conduct a family session in which potential participants will decide if they would like to take part in Group PM+. The research assistant will ask the potential participant what family member they would like present for the consent procedure. Potential participants also have the option of not including a family member in the consent process. With this model, the participant can gain support from their family in deciding if they would like to participate in the trial. In either phase of the consent process, it will be made clear that refusal to participate will not have an impact on any type of support they receive and that they will still be referred to local mhGAP-trained health workers and a counselor if needed.

#### Harms

The main risk is potential psychological distress among participants of the intervention arm depending on the type of interactions with other group members and group facilitator. Participants can stop their involvement in the trial at any point. All patients referred to mhGAP-trained health workers and TPO counselors are expected to be receiving quality clinical care and management of adverse events. Primary healthcare workers are supervised by a psychiatrist in Kathmandu who can provide information on medications and receive referrals for patients with worsening symptoms or other clinical concerns.

All changes in treatment resulting from adverse events or serious adverse events will be reported to the DSMC in Nepal. TPO Nepal is responsible for the data collection and storage and making data available to the DSMC, funders, and IRBs for audits when appropriate.

#### Post-trial care

Group PM+ facilitator training will be provided to those that attended CPSW basic training in the control arm after the trial. Though they will not be compensated through TPO, facilitators in the control arm could deliver Group PM+ sessions post-trial to their community with support from the local government. Primary healthcare workers will remain in the VDC and continue to provide mental health care for members of the community and Group PM+ trial participants.

### Dissemination

Findings from the feasibility study will be published in academic journals, disseminated through the Mental Health Innovation Network (www.mhinnovation.net), and reported to research funder (Office of U.S. Disaster Foreign Assistance/USAID). Findings will also be disseminated in Nepali and English to key stakeholders including district, provincial, and national government through reports and presentations. Authorship eligibility will comply with guidelines of the International Committee of Medical Journal Editors, with additional attention to recommendations for equitable representation of researchers from LMIC for academic authorship [41]. In keeping with transparency recommendations, data will be made publicly available after publication of primary analyses.

### Timescale

Participants for the Group PM+ trial will be recruited starting approximately 3 months after the initial CPSW training (see Table 5 for SPIRIT enrollment and assessment schedule). Group PM+ sessions will begin for those in the intervention arm within a maximum of 2 weeks after consent. Within these 2 weeks, baseline will be conducted for both arms. End line will be collected a week to a week and a half after the intervention is complete in the intervention arm and eight to eight and a half weeks after initial screening in the control arm. We anticipate that the trial will conclude by spring 2018.

**Table 5:** Schedule of enrollment, interventions, and assessments for Group PM+

	Study period		
PARTICIPANTS (*direct beneficiaries*)—participants of Group PM+ or control arm			
	Enrollment	Baseline	Follow-up
Timepoint	*− t*_1_	*t*_0_	*t*_1_
Enrollment			
Allocation	X		
Eligibility screen	X		
Informed consent	X	X	
Interventions			
PM+	X	X	X
Control	X	X	X
Assessments			
GHQ-12	X		X
WHODAS	X		X
AUDIT	X		
Suicidality	X		
PMPH		X	X
PHQ-9		X	X
PCL-5		X	X
PSYCLOPS		X	X
RTC		X	X
TEI		X	X
Heart-mind	X	X	X
MSPSS		X	X
Tension Checklist		X	X

## Discussion

The results of the feasibility trial will be used to determine whether we can move forward with the same procedures for the full trial in another region of Nepal. If there are qualitative or quantitative indicators of problems with feasibility and acceptability impacting recruitment, retention, randomization, fidelity, or safety, those relevant procedures will be modified. This is an external feasibility study, and therefore, data will not be carried forward from this study to the full trial. If significant modifications are needed, we will consider the need for an internal pilot in the context of the full trial [42].

There is growing evidence that interventions carried out by lay people from the communities are sustainable and feasible to implement on a broader scale, especially in low-resourced settings. As a brief trans-diagnostic intervention, PM+ has shown to be effective in reducing depression symptoms and improving people’s functioning in Pakistan and Kenya. If Group PM+ in Nepal is shown to be feasible and effective, this would provide evidence to scale-up within the country and would have implications for other low-resourced settings.

## Trial status

The trial is open and recruiting as of December 17, 2017. The protocol was last verified 22 January 2018. Subsequent protocol modifications will be reported to funders, IRBs, and registered with ClinicalTrials.gov.

## Abbreviations

AUDIT: Alcohol Use Disorder Identification Test
CMD: Common mental disorders
c-RCT: Cluster randomized controlled trial
ENACT: Enhancing Assessment of Common Therapeutic Factors
EUC: Enhanced usual care
LMIC: Low- and middle-income countries
mhGAP: Mental health Gap Action Programme
PHQ: Patient Health Questionnaire
PRIME: Programme for Improving Mental Health Care
TPO: Transcultural Psychosocial Organization Nepal
WHO: World Health Organization
WHODAS: World Health Organization Disability Assessment Scale
